# The Combination of Stent and Antiplatelet Therapy May Be Responsible of Parenchymal Magnetic Susceptibility Artifacts after Endovascular Procedure

**DOI:** 10.3390/tomography7040066

**Published:** 2021-11-13

**Authors:** Fanny Bourhis-Guizien, Brieg Dissaux, Grégoire Boulouis, Douraied Ben Salem, Jean-Christophe Gentric, Julien Ognard

**Affiliations:** 1 Department of Radiology, University Hospital of Brest, 29609 Brest, France; fanny.bourhis@chu-brest.fr (F.B.-G.); brieg.dissaux@chu-brest.fr (B.D.); douraied.bensalem@chu-brest.fr (D.B.S.); jean-christophe.gentric@chu-brest.fr (J.-C.G.); 2 Western Brittany Thrombosis Study Group GETBO EA3878, 29609 Brest, France; 3 Neuroradiology Unit, Department of Radiology, Saint-Anne Hospital, INSERM UMR 894, 75674 Paris, France; gregoireboulouis@gmail.com; 4 Laboratory of Medical Information Processing, LaTIM INSERM UMR 1101, 29200 Brest, France

**Keywords:** intracranial aneurysm, intracranial hemorrhage, magnetic resonance imaging, stents, magnetic susceptibility artifacts

## Abstract

The aim was to assess the occurrence of magnetic susceptibility artifacts (MSA) following endovascular treatment of intracranial aneurysm by stent using susceptibility weighted imaging (SWI). Imaging and clinical data of 46 patients who underwent stent placement in the case of intracranial aneurysm endovascular treatment (S-Group) were retrospectively analyzed and compared to a control group (C-Group) in which 46 patients had coiling alone. The mean number of MSA was higher in the S-group than in the C-group on postprocedural SWI sequence (8.76, 95%CI [5.76; 11.76] vs. 0.78 [0.32; 1.25], respectively, *p* < 0.001) with a higher frequency of the appearance of MSA also in the S-group (78.26% vs. 21.74% in the C-group, *p* < 0.001). In the S-group, in the vascular territory of the treated artery, there was a higher number of MSA than in other vascular territories (mean of 5.18 [3.43; 6.92] vs. 3.08 [1.79; 4.36], *p* = 0.001). An odds ratio (OR) of 20.98 [5.24; 83.95] suggested a higher proportion of onset of MSA in the S-group than in the C-group (*p* < 0.001). The appearance of MSA after a treatment by stenting for intracranial aneurysm in patients under antiplatelet therapy was common, particularly in the treated artery territory.

## 1. Introduction

Endovascular coil embolization is a widely performed treatment modality for intracranial aneurysms. This technique attempts to exclude the aneurysm from the intracranial circulation by filling the sac with coils [1]. This technique is safe and effective with a risk of early rebleeding estimated as 1.8% in the first year, and the degree of aneurysm occlusion is a strong predictor of this risk [2]. Coil treatment does not require antiplatelet therapy, which is an advantage, especially in the hemorrhagic phase. One of the major problems of coils is the rate of recanalization of the aneurysmal sac [3].

This is why embolization techniques using stents (including flow diverters, FDs) are increasingly used, especially for difficult-to-treat aneurysms [4,5,6] (i.e., giant aneurysms, fusiform aneurysms, blood blister-like aneurysms, and wide neck aneurysms). The risk of recanalization after treatment with stent is lower compared to treatment with coils alone [4,6,7,8].

Stenting requires a transient dual antiplatelet therapy to avoid the formation of intra-stent thrombosis [6,9]. Some authors have reported both early and delayed thrombotic events [9,10], and therefore the duration of this dual antiplatelet therapy is discussed between teams.

The need for a dual antiplatelet therapy limits stenting indications in the hemorrhagic phase [11] and contributes to the morbi-mortality of this technique considering rare hemorrhagic complications such as intracranial hematoma [12,13,14]. A review of the literature by Rouchaud et al. [14] found 101 cases of intraparenchymal hemorrhage following the placement of FDs unrelated to rebleeding. In 80% of these cases, the intraparenchymal hemorrhages were distributed in the territory of the treated artery. The pathophysiology remains controversial. To our knowledge, only two studies have shown the appearance of magnetic susceptibility artifacts (MSAs) in the territory of the stented artery [15,16]. Some of these MSAs were in the same locations of early ischemic lesion, suggesting that these MSAs would correspond to hemorrhagic transformation (microbleeds) [17]. In addition, previous studies have shown that antiplatelet therapy and the presence of microbleeds are both risk factors for intracerebral hemorrhage [17,18].

The aim of this study was to assess the occurrence of MSA following endovascular treatment of intracranial aneurysm by stent using susceptibility weighted imaging (SWI).

## 2. Materials and Methods

### 2.1. Patients

Between June 2015 and June 2018, we retrospectively reviewed at our center the demographic and radiological data of consecutive patients (age, sex, aneurysm localization, type and duration of antiplatelet therapy, history of previous sub-arachnoid hemorrhage (SAH) in the case of retreatment, date of first and follow-up MRI examinations) treated by stent (including FDs) to constitute the S-group (initial *n* = 74).

Patients followed only by CT angiography or catheter angiography were excluded (*n* = 2). Patients treated during the hemorrhagic phase were excluded (*n* = 3). SWI sequences must have been repeated at least one time during the imaging follow-up, which had to be done on the same scanner, if not patient were excluded (*n* = 11). Patients previously receiving a long term anticoagulation or antiplatelet therapy were excluded (*n* = 9). Patients must have received only one stent during all the follow-up. Patients must have received the center standardized antiplatelet therapy during the center standardized follow-up, if not, they were excluded (*n* = 3). The S-group was finally composed of 46 patients.

A one for one control group was also retrospectively constituted, minimizing the age and sex differences of all patients between both groups. The same data were recorded keeping the same exclusion criterion for these patients, which had to be treated solely by coils (C-group, *n* = 46).

The follow-up of the last patient included was achieved in June 2021.

### 2.2. Antiplatelet and Anticoagulation Therapy

All patients treated with stent (S-group) received a dual antiplatelet therapy for 6 months (Aspirin 75 mg once a day and Ticagrelor 90 mg twice a day) and a simple antiplatelet therapy (Aspirin 75 mg once a day) for life. 

No patient treated with coils (C-group) had long term antiplatelet therapy. In this last group, the retrospective design did not permit to aggregate sufficient data on the exact peri procedural management of antiplatelet. But, the standard protocol in our center is to deliver a unique loading dose at the end of the procedure (250 mg of Aspirin). 

Per procedure management of preventive anticoagulation in our center is standardized as a loading weight-dependent dose of heparin, and followed by bolus injections to maintain a TCA ratio between 2 and 2.5 during the endovascular procedure only. 

### 2.3. Imaging and Clinical Follow-up

The standard imaging follow-up scheme in our center after an endovascular treatment consisted of an MRI examination at three to six months, 12 months, 24 months, and 36 months (+/−3 months for annual FU). A clinical examination was conducted in the same week at each date of the imaging follow-up. 

### 2.4. MRI Sequences 

All scans were performed on a three Tesla MRI scan (Achieva Dstream, Philips, The Netherlands). The SWI volumetric axial acquisitions were realized thanks to Multishot Fast Field Echo Plannar Imaging (FFE-EPI) with the following parameters: Field Of View: 220 ∗ 250 ∗ 150 mm; Acquisition Voxel size: 0.55 × 0.55 × 1 mm; Reconstructed Voxel size: 0.29 × 0.29 × 0.5 mm; SENSE factor: 2 (Phase) and 1.6 (Frequency); Echo Time: 29 ms; Relaxation Time: 55 ms; Flip angle: 17°; EPI Factor: 15; Time duration: 3 min 37 s. Number of Signal Average: 2; Bandwidth: 22 Hz (Water Fat Shift: 19.4 pixels). For each MRI examination, the standard protocol also contained these sequences: 3D T2 weighted FLAIR, axial diffusion weighted imaging (DWI), and 3D Time of Flight. 

### 2.5. Reading

Two radiologists (one junior and one senior) read each MRI independently, each time evaluating the presence or absence of MSA, a count of the total number of MSA and the number of MSA in the territory of the treated artery. These readers were blinded to patient administrative, clinical, or other previous radiological data. A senior reviewed the clinical data and other MRI sequences to report eventual ischemic or symptomatic lesion. 

### 2.6. Statistics

Statistical analysis was performed using StataMP 13.0 (StataCorp, College Station, TX, USA). Quantitative variables were described using mean and the 95% confidence interval (CI). The test for normal distribution was Shapiro–Wilk. Qualitative and binary variables were described using frequency. Mean between groups (C-group/S-group; FD and non-FD stents) were compared using the Wilcoxon rank sum test or *t*-test. Qualitative and binary variable distributions among groups were tested using Chi-square or Fisher exact test. Agreement between observers was calculated through the Kappa for binary responses and intraclass correlation coefficient for quantitative ones. Kappas were interpreted according to Landis and Koch. Association between the appearance of MSA and the use of stent was assessed using multivariate logistic regression, reporting an odds ratio (OR), with 50 bootstrap replications. As a way to determine the delay of occurrence of MSA, their appearance were considered as an event in a survival dataset using time between corresponding date and the date of the first examination, and analyzed using the Kaplan–Meier estimate failure function. A *p* value of less than 0.05 was used to denote a statistical significance.

## 3. Results

### 3.1. Population and Follow-Up

In the S-group, 21/23 (91.3%) of flow diverters used were the Pipeline Embolization Device (Medtronic, Minneapolis, MN, USA), and 2/23 (8.7%) were Silk+ (Balt Extrusion, Montmorency, France). The other stents (non-FD) were 13/23 (56.5%) Neuroform Atlas (Stryker Neurovascular, Fremont, CA, USA) and 10/23 (43.5%) Leo (Balt Extrusion, Montmorency, France).

Patients underwent a mean number of 3.57 scans in the S-group and a mean number of 4.80 in the C-group (*p* < 0.001). This number included a pretreatment scan for all cases in both groups. All patients of the S-group had 3–6 months follow-up MRI, 40 (86.96%) had 12 months follow-up, 37 (80.43%) had 24 months follow-up, and 46 (100%) had 36 months follow-up. All patients in the C-group had all of these scans. The follow-up duration was longer in the C-group (mean of 1095.54 days [1094.33; 1096.75]) than in the S-group (mean of 940.19 days [919.04;961.35]), *p* < 0.001). Fifty percent of the stents used in the S-group were FD (23/46). The follow-up duration was not different in the flow diverter sub-group (of the S-group) (mean of 972 days [952.47; 991.53]) than in the non-flow diverter one (mean of 908.39 days [890.96; 925.82], *p* = 0.206). Study population characteristics are reported in Table 1. 

### 3.2. Interobserver Agreement 

An almost perfect agreement between observers in the detection of MSA was noticed: Kappa = 0.91 with 95% CI [0.82; 1.00] and in the MSA count: intraclass correlation coefficient (ICC) = 0.93 [0.89; 0.95].

### 3.3. Clinical Outcome

According to the review of radiological and clinical data, none of the MSA were symptomatic. These MSA findings did not change any dual antiplatelet protocol. There were no abnormalities of signal on 3D T2 FLAIR weighted imaging around MSA, and a study of DWI revealed no recent ischemic lesion in these areas.

### 3.4. MSA Analysis

All described MSAs were described in the phase imaging as having the same behavior as microbleeds, which excluded mimics such as microdissections, microaneurysms, microcalcifications, and arteriolar pseudocalcifications. In addition, the study of all available sequences described a restricted blooming artifact. 

There was no difference between the C-group and the S-group in the number of MRI with MSA realized before treatment (0% and 8.5%, respectively, *p* = 0.565).

For all patients, the last follow-up MRI depicted the maximum number of MSA compared to previous scans (when there was MSA, and when they number increased). Thereby, in all cases, this last examination was taken to calculate the number of MSA for each patient. 

In the S-group, considering the 35 pretreatment scans (76%), the mean number of MSAs was higher after endovascular treatment than before treatment (0.55 [0.06; 1.04] vs. 8.26 [5.48; 11.04], respectively). This comparison was not realized in the C-group because of the fewer numbers (four) of available pretreatment MRI.

The mean number of MSA was higher in the S-group than in the C-group on postprocedural SWI sequence (8.76 [5.76; 11.76] vs. 0.78 [0.32; 1.25], respectively, *p* < 0.001) with a higher frequency of appearance of MSA also in the S-group (78.26% vs. 21.74% in the C-group, *p* < 0.001) (Table 2). 

There was no difference in the number of MRI in which an appearance of MSA was depicted between patients treated by the non-FD stent and patients treated by FD (19 (82.61%) vs. 17 (73.91%), *p* = 0.722). The type of non-FD stent did not influence the appearance of MSA (*p* = 0.618); this last analysis was not realized for FD stent because of the high prevalence of Pipeline in the study.

**Figure 1:**
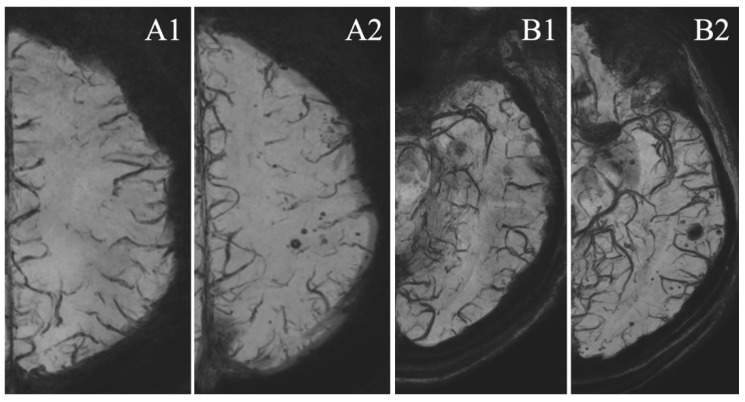
MSA appearance. Axial ten millimeters minimum intensity projection (Mini-IP) reconstructions of magnetic resonance imaging (MRI), susceptibility weighted imaging (SWI) sequence of the same patient. (**A1**,**B1**) Pretreatment scans, (**A2**,**B2**) follow-up at 36 months after treatment by stent (flow diverter, FD) of a left posterior communicating artery aneurysm. Notice the preferential disposition of MSA in the territory of the stented artery (left internal carotid artery).

In the S-group, in the vascular territory of the treated artery, there was a higher number of MSA than in other vascular territories (mean number of 5.18 [3.43; 6.92] MSA vs. 3.08 [1.79;4.36], *p* = 0.001) (Figure 1).

This rate for preferential territorial appearance was neither different when comparing the S-group and C-group (*p* = 0.319), nor when comparing the non-flow diverter subgroup and the flow diverter one (*p* = 1.000) (Table 2).

A logistic multivariate analysis introduced in the regression variables such as age, sex, follow-up duration, localization, and previous SAH showed a link (*p* = 0.033, Pseudo-R2: 23.26%) between the type of endovascular treatment and MSA appearance on SWI imaging follow-up (*p* < 0.001), with a calculated odds ratio (OR) of 20.98 [5.24; 83.95], suggesting a higher relative risk of onset of MSA in the S-group than in the C-group (Table 3). 

A survival analysis considered the appearance of at least one MSA as an event. In the S-group, the median probability of MSA appearance was reached in 112 days, and the first and third quartile in 96 and 545 days, respectively. 

## 4. Discussion

An occurrence of MSA after endovascular treatment of an intracranial aneurysm by stent or coils was depicted, and this appearance was higher in patients treated by stents, which is in agreement with the results of two previous studies: McGuiness et al. [16] demonstrated the presence of MSA following treatment with FD in 38% of cases (eight cases out of 21) on MRI realized early after the procedure. Nakae et al. [15] showed the occurrence of MSA at six months after treatment with FD or stent in 36.7% of cases and 11.1% of cases, respectively. MSA are artifactual by nature and likely to depict the presence of microscopic blood residues with yet unclear pathophysiology in this specific context, but it is likely that these blood residues represent hemorrhagic transformation of microinfarcts, as in the case of bleeding prone sporadic microangiopathy [19]. The increasing number of MSAs suggests active microvascular insults with the formation of micro-infarcts (with hemorrhagic transformation potentiated by antiplatelet therapy) who are a risk factor of hemorrhage, higher than microbleeds themselves in the context of microbleed prone diseases.

A higher number of MSA in the S-group than in the C-group was found, which suggests that the stent is indeed involved, but also the antiplatelet treatment. Effectively, this dual antiplatelet therapy taken for six months, and then simple antiplatelet therapy for life, is a risk factor for intracerebral hemorrhagic lesions [20]. Vernooij et al. [18] showed that patients under antiplatelet therapy had more microbleeds than untreated patients (OR = 1.71). Wong et al. [17] showed that antiplatelet therapy was a risk factor for intracranial hematoma.

This occurrence of MSA was frequent, especially in the S-group, because they were found in about 80% of the MRI. The SWI was used to depict MSA, and it is known to provide a high sensitivity to detect these artifacts [21]. Even if the MSA count could have been “overestimated” due to the sensitivity of this last MRI sequence, the fact remains that an almost perfect agreement between observers has been found. This reinforces the presumption of the reality of the phenomenon. 

This high frequency was not different between FD-treated patients and others in the S-group, which is in disagreement with the study conducted by Nakae et al., who found a higher number of MSA in patients treated with FD. The two previous studies showed that some of these MSAs corresponded to the same locations as ischemic lesions detected on diffusion sequences realized early after the endovascular procedure: in the Nakae study, 77.8% of cases with FD treatment and 100% of cases with other stents: these MSA could therefore correspond to hemorrhagic transformation of early ischemic lesions, the occurrence of which is frequent after endovascular aneurysm treatment whether by coils, stents, or FD [4,15,22,23].

Knowing that the FD are at higher risk of thromboembolic events than other stents (15), this inconsistent result could be explained by the type of antiplatelet treatment: in our study, patients were all treated by ticagrelor and aspirin, while in the Nakae study, patients were treated mainly with clopidogrel and aspirin. Ticagrelor is more effective in preventing the rate of thromboembolic events [24] because there are many hyporesponders for clopidogrel [25], but causes more hemorrhagic complications [26]. The type of antiplatelet agent and its management could explain why the number of MSA was not different between FD and the stent in this study, but could also lead to “avoiding” more occurrence of these MSA, if it appears necessary. This may also confirm that the occurrence of MSA is multifactorial. 

In another step, a higher number of MSA in the vascular territory of the treated artery by stent was noticed. This last result is consistent with the results of Nakae et al. and McGuiness et al. and several studies have also shown that the occurrence of ischemic micro-lesions following stenting occurred mainly in the territory of the treated artery (in 91.7% of cases (44 out of 48) in the study of Hahnmann [21] and in 100% of the cases in the study of Heller [27]). One explanation is that the appearance of these MSAs is related to the formation of micro-emboli by the aggregation of platelets in contact with the metal of the stent with distal occlusion of the arteries in the vascular territory and formation of ischemic lesions followed by secondary hemorrhagic transformation. However, the results from McGuiness et al. and Nakae et al. show that some MSA did not correspond to early ischemic lesion (McGuiness et al.: in 38% of cases the MSA were in the stent territory but did not correspond to ischemic abnormalities. Nakae et al.: in the FD-treated group, 32.2% of the MSA did not correspond to ischemic micro-lesions). Therefore, another hypothesis would be that stent placement would lead to hemodynamic changes by decreasing the flow in systole and diastole and reduces arterial compliance, which would reduce the Windkessel effect [28] with consequent cerebral hyperperfusion complicated with distal bleeding micro-lesions. Chiu reports a case of distal cerebral hyperperfusion in the territory of the stented artery following the placement of a FD [29]. It is also possible that these MSA correspond to the emboli of used equipment such as ferromagnetic material or polyvinylpyrrolidone (PVP), a substance that is commonly used in the coatings of interventional devices [30]. This last hypothesis supports the results that MSA tended to appear the most in the first three months but continued to appear during the follow-up.

This study had limits of a monocentric one. The retrospective design also resulted in some heterogeneity in the follow-up between the two groups, although a standardized design was chosen to keep patients in the study. As the occurrence of MSA seems to be multifactorial, the obvious limit is the lack of analysis on many factors such as device type, arterial blood pressure (baseline, during and after the procedure), different antiplatelet management (type and duration), and early ischemic changes on immediate postprocedural MRI. The number of patients did not permit an analysis with enough power multiple factors that could interfere with the treatment-related occurrence of MSA such as cardiovascular risk factors [31]. It would be interesting to carry out larger studies that control the previous variable and with magnetic susceptibility sequences conducted early after the procedure to detect MSA not related to ischemic foci, and therefore possibly related to hemodynamic changes. In order to distinguish the different etiologies above-mentioned, it may be appropriate in a future study on the subject to systematize MRI immediately after endovascular procedures. Long-term follow-up of these patients would also make it possible to evaluate the risk of late hemorrhagic complication or clinical deficit to see whether the findings described in this study may lead to events or if they are purely incidental.

## 5. Conclusions

The appearance of MSA after treatment by stenting for intracranial aneurysm in patient under antiplatelet therapy was common, particularly in the treated artery territory. The nature and exact cause of these MSA are uncertain and probably multifactorial, and as they may be microbleeds, the question arises of the delayed risk of intra-cerebral hemorrhage in these patients under antiplatelet therapy. 

## Figures and Tables

**Table 1:** Summary of the demographic, clinical, and radiological characteristics of the population.

Characteristics	S-Group (*n* = 46)	C-Group (*n* = 46)	*p*-Value
Age (years)	53.22 [49.69; 56.74]	55 [51.36; 58.64]	0.480
Sex (female)	33 (71.74%)	33 (71.74%)	1.000
History of SAH	20 (43.47%)	36 (78.26%)	0.001
*Localization*			
Anterior complex	4 (8.70%)	4 (8.70%)	0.003
Carotid and MCA	36 (78.26%)	22 (47.82%)	
Posterior circulation	6 (13.04%)	20 (43.48%)	
Flow diversion	23 (50%)	-	
Dual antiplatelet therapy duration (days)	185.1 [172;16; 182.07]	-	
*Details of the imaging survey*			
Number of scan (pretreatment and FU)	3.57 [3.32; 3.82]	4.80 [4.56; 5.04]	<0.001
Mean FU duration (days)	940.19 [919.04; 961.35]	1095.54 [1094.33; 1096.75]	<0.001
number of M3-6 FU	46 (100%)	46 (100%)	
number of M12 FU	40 (86.96%)	46 (100%)	
number of M24 FU	37 (80.43%)	46 (100%)	
number of M36 FU	46 (100%)	46 (100%)	

SAH: sub-arachnoid hemorrhage; M: months; FU: follow-up.

**Table 2:** Assessment of MSA in the different groups. Comparisons between the stent group (S-group) including non-flow diverter stent (non-FD stent) and FD ones (FD stent) and the coils group (C-group) in terms of the mean number of magnetic susceptibility artifacts (MSA) depicted in the last MRI of the follow-up. Appearance of MSA depicted when new MSA was noticed on a follow-up scan (only once for each patient) and territorial appearance of MSA depicting the preferential localization of previously appeared MSA in the arterial territory of the treated artery.

	S-Group (*n* = 46)	Non-FD Stent (*n* = 23)	FD Stent (*n* = 23)	C-Group (*n* = 46)	*p*-Value
Mean number of MSA	8.76 [5.76; 11.76]			0.78 [0.32; 1.25]	<0.001
		3.77 [2.09; 5.45]	7.78 [3.10; 12.46]		0.022
Appearance of MSA	36 (78.26%)			18 (21.74%)	<0.001
		19 (82.61%)	17 (73.91%)		0.722
Territorial appearance of MSA	34 (94.44%)			15 (83.33%)	0.319
		18 (94.74%)	16 (94.11%)		1.000

**Table 3:** Link of magnetic susceptibility artifacts (MSA) appearance with belonging in the S-group through the results of multivariate logistic regression. * Multivariate logistic regression, Bootstrap replication: 50, Pseudo-R2: 23.26%; *p* = 0.033.

	MSA Appearance: Multivariate Analysis *			
	OR	95% CI		*p*
S-group	20.98	5.24	83.95	<0.001
Age	1.04	0.98	1.11	0.165
Sex	1.68	0.47	5.98	0.423
Follow-up duration	1.00	0.99	1.00	0.081
Localization	1.97	0.68	5.71	0.211
Previous Sub-Arachnoid Hemorrhage	1.36	0.34	5.37	0.662

## Data Availability

The data presented in this study are available on reasonable request from the corresponding author.
